# ﻿A new species of the genus *Amolops* (Anura, Ranidae) from the Gaoligong Mountains, China

**DOI:** 10.3897/zookeys.1227.131357

**Published:** 2025-02-11

**Authors:** Yun-He Wu, Zhong-Bin Yu, Felista Kasyoka Kilunda, Chen-Qi Lu, Jia-Hua Li, Yun-Peng Li, Yi-Juan Shi, Jing Che

**Affiliations:** 1 Key Laboratory of Genetic Evolution and Animal Models, and Yunnan Key Laboratory of Biodiversity and Ecological Conservation of Gaoligong Mountain, Kunming Institute of Zoology, Chinese Academy of Sciences, 650223, Kunming, Yunnan, China Kunming Institute of Zoology, Chinese Academy of Sciences Kunming China; 2 Southeast Asia Biodiversity Research Institute, Chinese Academy of Sciences, 05282, Yezin, Nay Pyi Taw, Myanmar Southeast Asia Biodiversity Research Institute, Chinese Academy of Sciences Yezin Myanmar; 3 Kunming College of Life Science, University of the Chinese Academy of Sciences, 650204, Kunming, Yunnan, China University of the Chinese Academy of Sciences Kunming China; 4 Longyang Branch of Gaoligongshan National Nature Reserve, Baoshan Management Bureau, Baoshan, Yunnan 678000, China Longyang Branch of Gaoligongshan National Nature Reserve, Baoshan Management Bureau Baoshan China

**Keywords:** *Amolops viridimaculatus* group, diversity, new species, phylogeny, sympatric species, Yunnan Province

## Abstract

The Gaoligong Mountains lie at the intersection of three global biodiversity hotspots. In recent years, with the continuous deepening of fieldwork in the region, studies have increasingly indicated that the amphibian diversity of this region remains poorly understood. During herpetological surveys in 2023, a series of *Amolops* specimens were collected from the Gaoligong Mountains of Yunnan Province, China. The integrated results of morphological and molecular phylogenetic analyses indicate the presence of a separate and previously unknown lineage in the *A. viridimaculatus* group, which we herein confirm as a new species, *Amolops gudao* Yu, Wu, Lu & Che, **sp. nov**. Our discovery increases the number of *Amolops* species recorded in China to 59, and the total number of *Amolops* species to 86. The discovery of a new species in the Gaoligong Mountains further highlights the underestimated amphibian diversity in the region, emphasizing the need for continued fieldwork and research. Furthermore, *Amolops gudao* Yu, Wu, Lu & Che, **sp. nov.** exhibits obvious intraspecific variation in color patterns, a phenomenon also reported in several species within the *A. viridimaculatus* group, such as *A. viridimaculatus* and *A. kaulbacki*. Future studies on the taxonomy of *A. viridimaculatus* group should be careful with the use of color patterns as a diagnostic characteristic.

## ﻿Introduction

The torrent frogs of the genus *Amolops* Cope, 1865 are the most species-rich in the family Ranidae. Currently, *Amolops* contains 85 recognized species with a wide distribution from Nepal and northern India eastwards to southern China and southwards to Peninsular Malaysia (Frost 2024). The *A. viridimaculatus* species group, first proposed by Wu et al. (2020), is mainly distributed in the Himalayas, Gaoligong Mountains, extreme southern Yunnan, and northern Indo-Burma (Che et al. 2020; Frost 2024; Wu et al. 2024a). It is characterized by the following morphological traits: a large body size, absence of dorsolateral folds, distinct vomerine teeth, tarsal fold and tarsal glands absent, vocal sac absent in males, and nuptial pad present on the first finger in males (Wu et al. 2020; Jiang et al. 2021). Species within the *A. viridimaculatus* group have been extensively discussed by various authors (Jiang et al. 2021; Mahony et al. 2022; Saikia et al. 2022; Wu et al. 2020, 2024a). As a result, the most recent classification of *Amolops* listed 13 species in the *A. viridimaculatus* group.

The Gaoligong Mountains are a sub-range at the southwestern end of the Hengduan Mountains, situated in the watershed between the Salween River to the east and the Irrawaddy River to the west. It lies at the intersection of Myanmar and Southwest China’s Yunnan Province and Xizang Autonomous Region, a region where three globally significant biodiversity hotspots converge (Himalayas, Indo-Burma, and the mountains of Southwest China; Mittermeier et al. 2011). The region can be subdivided into seven climate zones; tropical, southern subtropical, mid-subtropical, northern subtropical, warm temperate, temperate, and cold temperate zones (Wu et al. 2024a). Consequently, it is home to a high diversity of species including amphibians. In recent years, several cryptic and novel amphibian species have been described (e.g., Liu et al. 2021; Lyu et al. 2023; AmphibiaChina 2024; Lee et al. 2024; Wu et al. 2021, 2024a, b; Yu et al. 2024). These findings suggest that the amphibian fauna in the region may still be diverse and largely underestimated. A recent herpetological survey at the Gaoligong Mountains, Yunnan Province, China, obtained a collection of some *Amolops* specimens. Subsequent studies, including molecular data and morphological comparisons, revealed that it is clearly distinct from its *Amolops* congeners. Therefore, we describe it herein as a *Amolops gudao* sp. nov.

## ﻿Materials and methods

### ﻿Sampling

**Figure 1.**
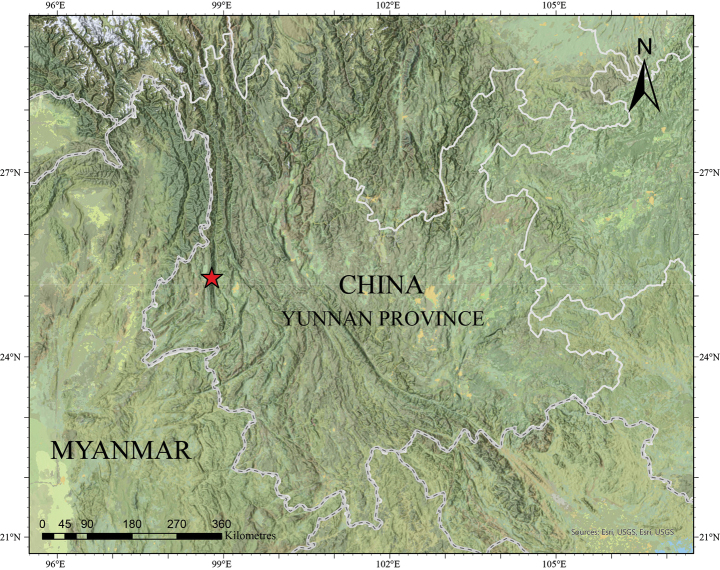
Map showing the type locality of *Amolops gudao* sp. nov.

During a field survey at Baihualing Village, Longyang, Baoshan, Yunnan Province, China, in July 2023 (Fig. 1). Four adult specimens (two males and two females) of *Amolops* were collected and photographed. Sex was determined by the presence of a nuptial pad and the presence of eggs in the abdomen, as observed via external inspection. After taking photographs, the frogs were euthanized using benzocaine. Liver tissue was taken from the specimens and preserved in 95% ethanol at -80 °C. The specimens were then fixed in 10% formalin for 24 hours and subsequently stored in 75% ethanol. All the newly collected specimens were deposited in the herpetological collection of the Museum of the Kunming Institute of Zoology (KIZ), Chinese Academy of Sciences (CAS). The research protocols were approved by the Ethics Committee of the Kunming Institute of Zoology, Chinese Academy of Science (IACUC no.: IACUC-OE-2021-07-001).

### ﻿Molecular data and phylogenetic analyses

Total genomic DNA was extracted from liver tissue stored in 95% ethanol using the standard phenol-chloroform extraction protocol (Sambrook et al. 1989). Three partial sequences of the mitochondrial 16S rRNA gene (16S rRNA), cytochrome oxidase subunit I (*COI*), and NADH dehydrogenase subunit 2 (*ND2*) genes were amplified and sequenced using the following primers: 16SAR (5’-CGCCTGTTTAYCAAAAACAT-3’) and 16SBR (5’-CCGGTYTGAACTCAGATCAYGT-3) (Kocher et al. 1989), ND2AR (5’-CAATGTTGGTTAAAATCCTTCC-3’) and NDBR (5’-AGGCTTTGAAGGCCTTTGGTC-3’) (Stuart et al. 2006), and Chmf4 (5’-TYTCWACWAAYCAYAAAGAYATCGG-3’), and Chmr4 (5’-ACYTCRGGRTGRCCRAARAATCA-3’) (Che et al. 2012). PCR amplification was performed in a 25μl reaction volume with the following cycling conditions: initial denaturation step at 94 °C for 5 min, 35 cycles of denaturation at 94 °C for 1 min, annealing at 55 °C for 16S rRNA, 50 °C for *ND2*, and 55 °C for *COI* for 45 s, extension at 72 °C for 1 min, and final extension at 72 °C for 10 min. PCR products were purified and sequenced in both directions by TSINGKE Biological Technology (Kunming, Yunnan). All the newly generated nucleotide sequences were initially assembled and manually edited using DNASTAR LASERGENE 7.1. The new sequences were deposited in the GenBank (Suppl. material 1).

To obtain the phylogenetic relationships among *A. viridimaculatus* group, homologous sequences of *A. viridimaculatus* group available in the NCBI GenBank were downloaded (Suppl. material 1). Our final dataset included 13 described species of *A. viridimaculatus* group. In cases of geographically widespread species, multiple samples from different localities were included. *Amolops wuyiensis* (Liu & Hu, 1975) and *A. spinapectoralis* Inger, Orlov & Darevsky, 1999 were chosen as outgroups based on a previous phylogenetic study (Wu et al. 2020). All sequences were aligned using MUSCLE 3.8 (Edgar 2004), then inspected by eye for accuracy and trimmed to minimize missing characters in MEGA 6 (Tamura et al. 2013).

The phylogenetic reconstruction was performed using Bayesian (BI) analyses and maximum likelihood (ML) methods. The best-fit model of evolution was determined using the Bayesian Information Criterion (BIC) by jModelTest 2.1.7 (Darriba et al. 2012). The HKY + I + G, HKY + G, and GTR + I + G models were chosen as the best-fit models following the Bayesian information criterion (BIC; Posada 2008) for 16S rRNA, *COI*, and *ND2*, respectively. The BI and ML analysis were implemented by the CIPRES web server (Miller et al. 2010). For BI analyses, the Monte Carlo Markov chain length was run for 10 million generations and sampled every 1000 generations with a burn-in of 25%. Convergence was assessed in Tracer 1.5 (Rambaut and Drummond 2009) based on having an average standard deviation of split frequencies (below 0.01) and ESS values (over 200). The ML analyses were performed using RAxML-HPC BlackBox 8.2.10 with 1,000 bootstrap replicates and using the standard bootstrap search (random seed value 12,345) under the GTR+gamma nucleotide substitution model (Stamatakis 2014). Uncorrected pairwise distances (*p*-distances) among species and individuals were calculated in MEGA 6 (Tamura et al. 2013).

### ﻿Morphology

Measurements were recorded to the nearest 0.1 mm with digital calipers by Zhong-Bin Yu following Fei et al. (2009). Measurements included: **SVL** (Snout-vent length): measured from tip of snout to vent; **HL** (head length): measured from tip of snout to jaw angle; **HW** (head width): measured as head width at its widest point; **SL** (snout length): measured from tip of snout to anterior corner of eye; **INS** (internaral space): measured as distance between nares; **IOS** (interorbital space): measured at narrowest point between eyelids on top of head; **NED** (nasal to eye distance): measured as distance from the anterior corner of eye to nostril center; **SND** (snout-nostril distance): measured as distance from nostril centre to the snout tip; **UEW** (upper eyelid width): maximum width of upper eyelid; **ED** (eye diameter): measured as the distance between corners of eye; **TD** (tympanum diameter): measured as maximal diameter of tympanum; **LAHL** (length of lower arm and hand): distance from elbow to the tip of the third finger; **HND** (hand length): measured as the distance from the proximal edge of inner metacarpal tubercle to the tip of third finger; **LAD** (diameter of lower arm); **FD1-4** (wide of the finger disk): greatest wide of disk on first to fourth finger; **HLL** (hindlimb length): from the tip of the fourth toe to vent; **FEM** (femoral length): measured from the cloaca to the knee; **TIB** (tibia length): measured as the distance from knee to heel; **FTL** (foot length): measured as the distance from proximal end of inner metatarsal tubercle to the tip of fourth toe; **IMT** (inner metatarsal tubercle length); **TD1-5** (wide of first toe disk), greatest wide of disk on first to fifth toe.

## ﻿Results

**Figure 2.**
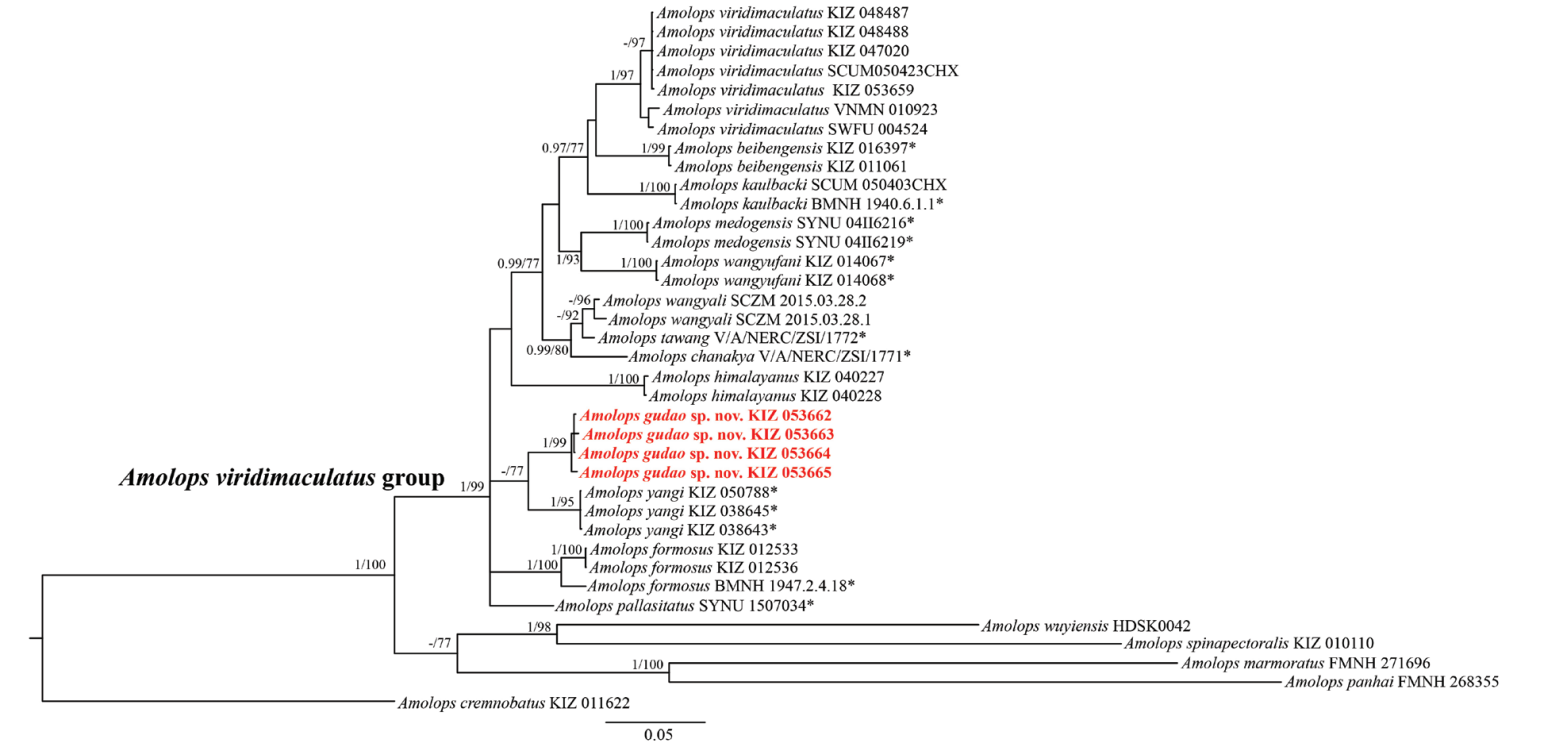
Phylogram of *Amolops* inferred from three mtDNA gene fragments (16S rRNA, *COI*, and *ND2*). Node values with Bayesian posterior probabilities (BPP) < 0.95/Bootstrap support (BS) < 70 are not shown. A “–” denotes Bayesian posterior probabilities (BPP) < 0.95 and bootstrap support (BS) < 70. New samples for the present study are indicated in red font. The “*” indicates that the sequences are derived from the holotype or paratype. Outgroup taxa not shown.

Our concatenated mtDNA alignment (*ND2*: 890 base pairs (bp), *COI*: 570 bp, 16S rRNA: 534 bp) contained 37 individuals with a total of 1994 bp, with 1137 conserved sites and 855 variable sites. Of the variable sites in the alignment, 627 were parsimony-informative (355 bp, 172 bp, and 100 bp, including the outgroup sequences). The BI and ML trees showed essentially identical topologies (Fig. 2). Although the basal relationships within the *A. viridimaculatus* group were not well resolved, most nodes present relatively robust support. The monophyly of *A. viridimaculatus* group was strongly supported and in agreement with the results of Wu et al. (2020, 2024a): BI = 1.00, ML = 99 (Fig. 2). Among these, four new samples from Baihualing Village nested within the *A. viridimaculatus* group, which strongly clustered into a lineage (BI = 1.00, ML = 99) and clustered with *A. yangi* with low support (Fig. 2). The maximal within-group genetic *p*-distance of the unknown lineage was 0.0% (Suppl. material 2). The genetic distance between the new population and other species of the *A. viridimaculatus* group ranged from 1.5% (with *A. yangi*) to 4.6% (with *A. chanakya*) for 16S rRNA (Table 2). It is comparable to the divergences among the nearest neighbor genetic distances of this group, which ranged from 0.4% (*A. wangyali* and *A. tawang*) to 6.2% (*A. himalayanus* and *A. wangyufani*) for 16S rRNA (Table 2).

Morphologically, our newly collected specimens can be distinguished from all named species of *A. viridimaculatus* group (details in the taxonomic account below), which can be reliably identified by relatively smaller body size, absent rictal gland, tibiotarsal articulation reaching the anterior corner of eye, and the vivid green ground coloration of dorsum, interspersed with irregular black spots (Suppl. material 3).

### ﻿ Amolops gudao

Taxon classificationAnimaliaAnuraRanidae

Yu, Wu, Lu & Che sp. nov.

905DE1FA-40A9-5D6F-B02C-7701B66A1414

https://zoobank.org/EA17281D-8F9C-49B8-AB66-790060AC1008

Figs 1, 2, 3, 4, 5

#### Type material examined.

***Holotype*** • Adult male (KIZ 053662), from Baihualing Village, Longyang, Baoshan, Yunnan Province, China (25.301208°N, 98.788815°E; elevation 1793 m a.s.l.), collected by Zhong-Bin Yu, Dong An, Tian-En Chen on 19, July, 2021. ***Paratypes*** • One adult male (KIZ 053663), two adult females (KIZ 053664 and KIZ 053665); collected at the same locality and with the same collection information as the holotype.

#### Diagnosis.

The new species is recognized as a member of the *A. viridimaculatus* group based on molecular phylogenetic analyses and can be distinguished from its groups by a combination of the following characters (Suppl. material 3): (1) medium body size (SVL 56.7–58.6 mm in males and 63.6–64.8 mm in females); (2) vomerine teeth developed, on two short oblique between choanae, “八”-shaped, almost equal in distance from each other as to choanae; (3) supratympanic fold indistinct; (4) true dorsolateral folds absent, discontinuous glandular dorsolateral fold from rear of eye to near vent present; (5) tongue cordiform, 1/2 notched posteriorly; (6) maxillary teeth developed; (7) circummarginal grooves present on tips of outer three fingers, absent on first finger; (8) inner metacarpal tubercle rounded, outer metacarpal tubercle indistinct; (9) tibiotarsal articulation of adpressed limb reaching the anterior corner of eye; (10) a black stripe below edge of the canthus rostralis extending from the nostril across the eyes, along dorsolateral glandular folds to near vent; (11) rictal gland absent; (12) iris distinctly bicolored, green in upper 1/4 and reddish brown in lower 3/4, black reticulations throughout; (13) vocal sac absent in males; (14) male with orange nuptial pad at the base of first finger.

#### Description of holotype.

**Figure 3.**
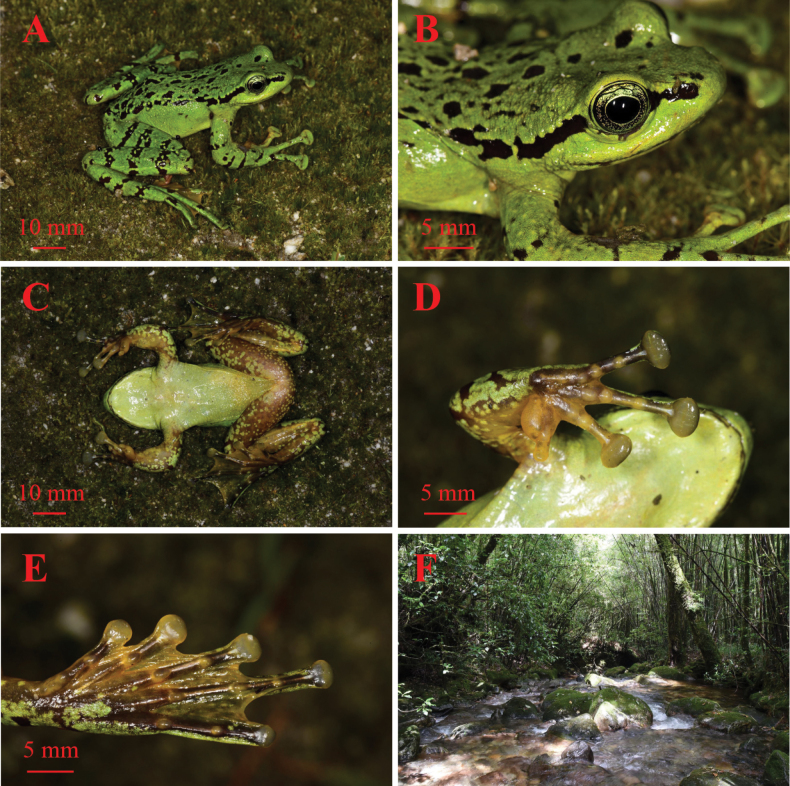
Views of the holotype KIZ 053662 in life **A** lateral view **B** lateral view of head **C** ventral view **D** ventral view of the hand **E** ventral view of foot **F** habitat. Photographs by Zhong-Bin Yu.

**(measurements in Table 1)**KIZ 053662, a medium-sized adult male (SVL 58.6 mm); body relatively robust; head slightly longer than wide (HL 18.2 mm; HW 17.7 mm); outline of the snout rounded in dorsal, ventral and lateral views, projecting beyond lower jaw, its length longer than diameter of eye (SL/ED 131.7%); loreal region concave and oblique; canthus rostralis distinct; interorbital space flat, shorter (IOS 4.9 mm) than upper eyelid width (UEW 5.2 mm) and internarial distance (INS 6.5 mm); nostril oval, laterally orientated, closer to anterior corner of eye (NED 3.8 mm) than tip of snout (SND 4.1 mm); tympanum indistinct, circular in shape, relatively small (TD/HL 13.7%), tympanic rim slightly elevated, indistinct; eyes relatively large (ED/HL 34.6%), slightly protuberant in dorsal view (Fig. 3A) and notably protruding in profile (Fig. 3B), eye diameter longer than snout length (SL/ED 131.7%); pupil horizontal (Fig. 3B); vomerine teeth developed, on two short oblique rows between choanae, “\/”-shaped, almost equal in distance from each other as to choanae; tongue attached anteriorly, cordiform, 1/2 notched posteriorly; choanae oval; maxillary teeth developed; indistinct tooth-like projection on anteromedial edge of mandible.

**Table 1.** Measurements (mm) of *Amolops gudao* sp. nov. The asterisk (*) indicates the holotype.

	*A. gudao* sp. nov.	*A. gudao* sp. nov.	*A. gudao* sp. nov.	*A. gudao* sp. nov.
Catalog No.	KIZ 053662*	KIZ 053663	KIZ 053664	KIZ 053665
Sex	Male	Male	Female	Female
SVL	58.6	56.7	64.8	63.6
HL	18.2	18.3	19.8	20.0
HW	17.7	17.7	20.0	20.0
SL	8.3	8.4	8.8	8.6
SL/HL	45.6%	45.9%	13.6%	13.5%
ED	6.3	6.1	6.6	6.1
ED/HL	34.6%	33.3%	33.3%	30.5%
SL/ED	131.7%	137.7%	133.3%	141.0%
IOS	4.9	5.4	5.4	5.0
UEW	5.2	4.6	5.2	5.7
INS	6.5	6.4	6.9	6.8
NED	3.8	4.2	4.0	4.2
SND	4.1	4.3	4.3	4.2
TD	2.5	1.8	2.0	1.9
TD/HL	13.7%	9.8%	10.1%	9.5%
LAHL	30.6	30.5	34.6	33.2
LAHL/SVL	52.2%	53.8%	53.4%	52.2%
HND	19.7	19.3	22.5	21.2
FD2	3.6	3.3	4.2	3.5
FD3	4.2	3.5	4.6	4.3
FD4	4.0	3.6	4.8	4.3
LAD	7.1	6.8	6.5	5.6
HLL	98.6	98.8	104.6	104.3
HLL/SVL	168.3%	174.3%	161.1%	164.0%
FEM	28.4	27.4	30.8	30.0
TIB	29.9	29.7	31.2	30.9
FEM/TIB	95.0%	92.3%	98.7%	97.1%
TIB/SVL	51.0%	48.3%	48.1%	48.6%
FTL	32.0	32.7	33.2	32.8
FEM/FTL	88.8%	83.8%	92.8%	91.5%
IMT	4.2	4.3	3.7	4.0
T	6.1	6.4	8.1	7.0
IMT/T	68.9%	67.2%	45.7%	57.1%
TD1	2.9	2.5	3.3	3.0
TD2	3.4	2.8	3.8	3.3
TD3	3.2	2.8	3.6	3.3
TD4	2.2	2.0	2.8	3.0
TD5	1.7	1.7	2.1	2.6

**Table 2.** Average uncorrected *p*-distances (percentage) among *Amolops viridimaculatus* group calculated from 16S rRNA gene sequences (below the diagonal, black font) and standard error estimates (above the diagonal, blue font). The ingroup mean uncorrected *p*-distances are shown on the diagonal.

ID	Species name	1	2	3	4	5	6	7	8	9	10	11	12	13
**1**	*Amolops viridimaculatus*	**0.2**	0.7	0.6	0.8	0.8	1.0	1.1	0.9	0.8	0.8	0.9	0.7	0.8
**2**	*Amolops kaulbacki*	2.3	**0.0**	0.7	0.8	0.9	1.0	1.1	1.0	0.8	0.7	0.8	0.7	0.9
**3**	*Amolops beibengensis*	1.8	2.1	**0.0**	0.8	0.8	1.0	1.2	0.9	0.9	0.8	0.9	0.8	0.9
**4**	*Amolops medogensis*	2.9	3.1	2.8	**0.0**	0.9	1.0	1.1	0.8	0.8	0.6	0.9	0.6	0.9
**5**	*Amolops wangyufani*	3.2	3.8	2.8	3.3	**0.0**	1.0	1.2	1.0	0.9	0.9	1.0	0.9	1.0
**6**	*Amolops formosus*	4.3	4.1	4.6	4.1	4.4	**0.0**	1.0	1.0	0.9	1.0	1.0	1.0	1.0
**7**	*Amolops himalayanus*	5.1	4.9	5.4	4.9	6.2	4.4	**0.0**	0.9	0.8	1.1	1.1	1.1	0.9
**8**	*Amolops pallasitatus*	3.6	3.8	3.3	3.1	4.1	3.8	3.6	–	0.8	0.9	0.9	1.0	0.9
**9**	*Amolops yangi*	2.7	2.8	3.3	2.8	3.6	3.3	3.1	2.6	**0.0**	0.7	0.9	0.7	0.6
**10**	*Amolops wangyali*	2.5	2.2	2.7	1.9	3.5	4.2	5.0	4.0	2.4	**0.3**	0.7	0.3	0.9
**11**	*Amolops chanakya*	3.6	2.8	3.3	3.1	4.6	3.8	5.1	3.6	3.1	1.9	–	0.7	1.1
**12**	*Amolops tawang*	2.2	2.3	2.8	2.1	3.6	3.8	5.1	4.1	2.6	0.4	2.1	–	0.9
**13**	*Amolops gudao* sp. nov.	2.7	3.3	3.3	3.3	3.6	4.4	3.6	3.1	1.5	3.5	4.6	3.6	**0.0**

Forelimbs moderately long and robust, forearms significantly enlarged relative to upper arms; forelimb and hand length (LAHL 30.6 mm) longer than 1/2 body size (LAHL/SVL 52.2%); relative length of fingers: FI < FII < FIV < FIII; tips of all four fingers expended into discs, disc on finger III approximately equal to 1/2 snout length (FD3 4.2 mm; FD3/SL 50.6%); circummarginal grooves present on tips of outer three fingers, absent on first finger; subarticular tubercles prominent and oval, formula 1, 1, 2, 2; supernumerary tubercles present at the base of each finger; webbing between fingers absent; narrow lateral fringes of fingers III and IV; inner metacarpal tubercle rounded, outer metacarpal tubercle indistinct (Fig. 3D).

Hindlimbs long and robust, almost three times longer than the forearms and nearly two times longer than body length (LAHL/HLL 31.0%; SVL/HLL 59.4%); femoral length shorter than the tibia length (FEM/TIB 95.0%) and the foot length (FEM/FTL 88.8%); tibiotarsal articulation of adpressed limb reaching the anterior corner of eye when hindlimb is stretched alongside the body; the heels slightly overlapping when the tibias are perpendicular to the body axis; relative toe lengths: TI < TII < TIII < TV < TIV; toes with narrow lateral fringes, toe IV and postaxial side of toe V relatively wider; tips of all toes expanded into discs (TD1 2.9 mm; TD2 3.4 mm; TD3 3.2 mm; TD4 2.2 mm; TD5 1.7 mm), toe discs relatively smaller than those of fingers; toe discs with circummarginal grooves; toes fully webbed except for fourth toe, in which web reaches beyond distal subarticular tubercle; subarticular tubercles oval and distinct, formula 1, 1, 2, 3, 2; supernumerary tubercles absent; inner metatarsal tubercle elongate (IMT/TI 68.9%), outer metatarsal tubercle absent (Fig. 3E).

Skin on dorsal surface of head, body and limbs smooth; flank of body, ventral surface of head and limbs, throat, chest and abdomen relatively smooth; temporal region without any tubercles; supratympanic fold indistinct; true dorsolateral folds absent, but discontinuous series of glands along dorsolateral junction of body (dorsolateral glandular folds) present, extending from rear of eye to near vent; posterior angle of the jaw with dense tubercles; rictal gland absent (Fig. 3).

#### Color of holotype in life.

The ground coloration of dorsum vivid green, interspersed with irregular black spots; ventral surface of the head, throat, chest and belly mostly yellow-green, posterior abdomen with dense yellow dots; ventral surface of thighs, tibia, and tarsus brown, scattered with green spots and blotches; lateral surfaces of the body vivid green; a black stripe below edge of the canthus rostralis extending from the nostril across the eyes, along dorsolateral glandular folds to near vent; the dorsal surfaces of limbs with irregular black transverse bands, mottled with yellow-green dots, and the bands much more distinct on the hindlimbs, bands relatively incomplete on the forelimbs; iris distinctly bicolored, green in upper 1/4 and reddish brown in lower 3/4, black reticulations throughout; foot webbing brown; ventral surface of outer three fingers discs and outer three toes discs, metatarsal tubercle, subarticular tubercles of toes gray; ventral surface of first finger disc, inner two toes discs, subarticular tubercles and supernumerary tubercles of fingers orange yellow (Fig. 3).

#### Color of holotype in preservative.

**Figure 4.**
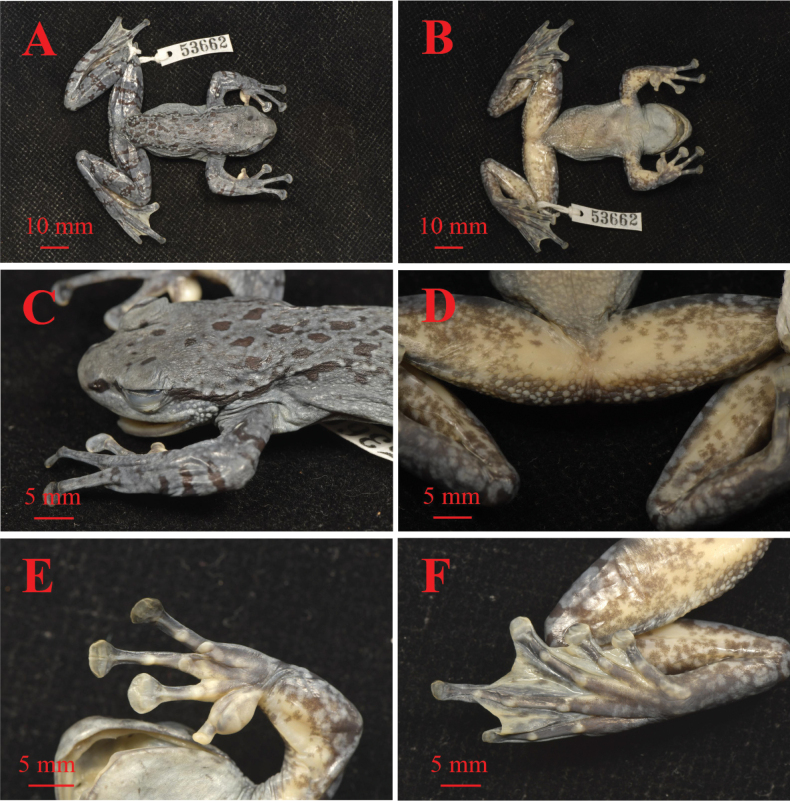
Views of the holotype KIZ 053662 in preservative **A** dorsal view **B** ventral view **C** lateral view of the head **D** ventral view of thigh **E** ventral view of hand **F** ventral view of foot. Photographs by Zhong-Bin Yu.

After one year of storage in ethanol, dorsum metallic blue, with small scattered blackish spots; transverse cross-bars on dorsal surfaces of hands, shanks, tarsus, and feet still clear and turning black; a black stripe below edge of the canthus rostralis extending from the nostril across the eyes, along dorsolateral glandular folds to near vent still clear; chest, throat and ventral surface of head fading to dark gray; ventral surface of limbs fading to cream-yellow, scattered with brown pigmentations; abdomen brown, gray around abdomen; posterior angle of the jaw and posterior surface of thigh around vent with dense tubercles more distinct; digit tips, nuptial pads, supernumerary tubercles, subarticular tubercles, and metatarsal tubercle fading to cream-yellow or grayish-white; foot webbing cream-yellow (Fig. 4).

#### Secondary sexual characters.

Adult female specimens have larger body sizes than adult male specimens (SVL 63.6–64.8 mm vs 56.7–58.6 mm, Table 1). Adult males possess orange nuptial pads covering the dorsal surface of base of the first finger; absence of vocal sacs in males. Forearms of male are slightly enlarged relative to the upper arms (Fig. 3).

#### Morphological variation.

**Figure 5.**
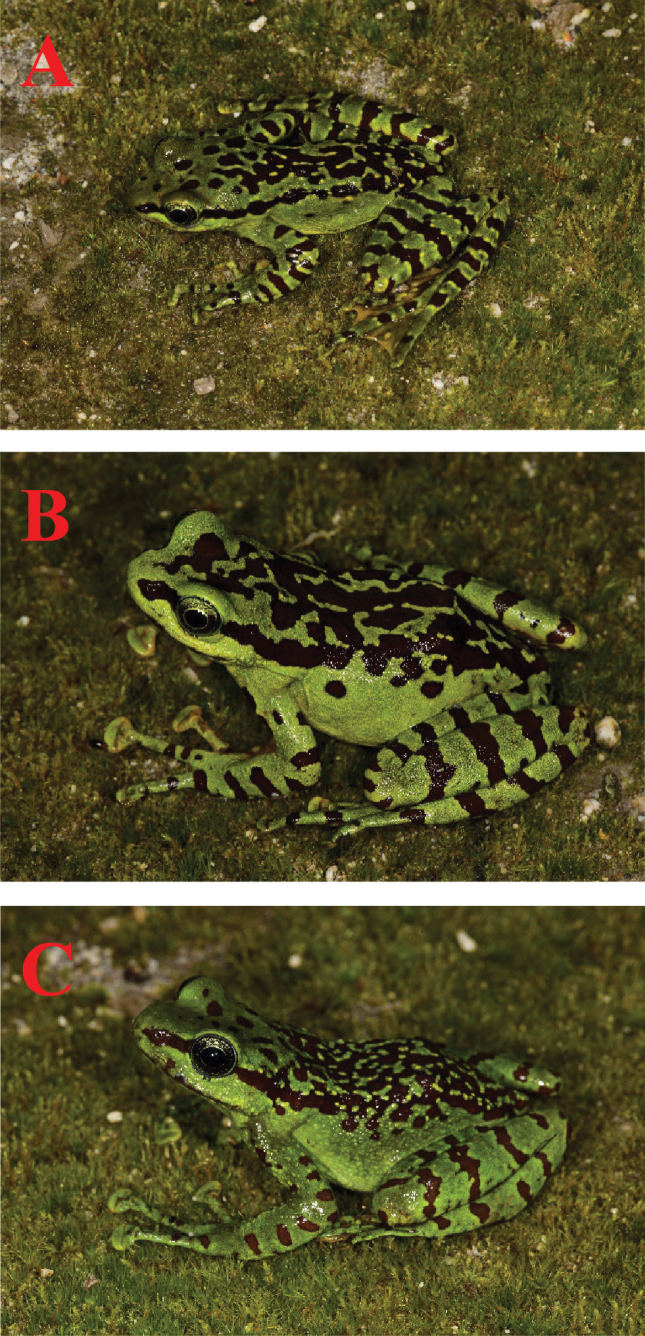
Morphological variation. Photographs of different individuals of *Amolops gudao* sp. nov. from the type locality, Baihualing, Longyang, Yunnan, showing **A**KIZ 053663 (male) **B**KIZ 053664 (female) **C**KIZ 053665 (female). Photographs by Zhong-Bin Yu.

The overall morphology of paratypes agrees with the holotype description with the following exceptions. KIZ 053663 (male): The ground coloration of dorsum vivid green, with larger and more irregular black markings mottled on the dorsal area, scattered with a few yellow spots; complete transverse cross-bars on dorsal surfaces of limbs, cross-bands with distinct yellow edge. KIZ 053664 (female): compared to holotype and paratypes, larger irregular black markings mottled on dorsal area, scattered with a few yellow spots; complete transverse cross-bars on dorsal surfaces of hindlimbs, cross-bands without distinct yellow edge; flanks present two black specks. KIZ 053665 (female): compared to other holotype and paratypes, smaller irregular black markings mottled on dorsal area, connected to form reticulations throughout; upper lips with three dark vertical bars; flanks mottled with seven black specks; dorsal surfaces of hindlimbs without complete transverse cross-bars, but scattered with some black specks; complete transverse cross-bars on dorsal surfaces of hindlimbs (Fig. 5).

#### Distribution.

Currently, this species is only known from Baihualing village, Longyang, Yunnan, China.

#### Ecology and habitat.

This nocturnal species inhabits wide and swift mountain streams. There were many large stones covered with moss in the stream. The frog was observed squatting on big rocks in the streams or on banks during our survey from 21:00 to 24:00 in July. When disturbed, it immediately jumps into the water. The vegetation on both sides of the stream was dense, surrounded by broad-leaved forests and bamboo forests. The adults were only found on July 19. The adult males displayed orange nuptial pads and the females were gravid during specimen collection. The tadpoles of this species were found on July 8 at the collection site. Therefore, we speculate that the breeding season of this species occurs around July-August. This species is sympatric with *A. viridimaculatus* (Jiang, 1983) and its congeners. The new species was also found in sympatry with *Leptobrachella alpina* (Fei, Ye & Li, 1990), *Oreolalax jingdongensis* (Ma, Yang & Li, 1983), *Bufo tuberospinius* (Yang, Liu & Rao, 1996), *Boulenophrys lushuiensis* (Shi, Li, Zhu, Jiang, Jiang & Wang, 2021), *Leptobrachium huashen* Fei & Ye, 2005, *Nanorana yunnanensis* (Anderson, 1879), and *Zhangixalus burmanus* (Andersson, 1939).

#### Comparisons.

The new species was compared with the other species of the *A. viridimaculatus* species group (Suppl. material 3; Andersson 1938; Biju et al. 2010; Nidup et al. 2016; Qi et al. 2019; Che et al. 2020; Mahony et al. 2022; Saikia et al. 2022; Hou et al. 2023; Wu et al. 2024a).

*Amolops gudao* sp. nov. is significantly different from *A. wangyali* by smaller body size, male SVL 56.7–58.6 mm and female SVL 63.6–64.8 mm (vs SVL 71.4–76.7 mm in males and 80.5–89.6 mm in females), rictal gland absent (vs a distinct patch of rictal glands at rear of jaw on either side), iris distinctly bicolored, green in upper 1/4 and reddish brown in lower 3/4, black reticulations throughout (vs pupil with near continuous pale metallic green border, remaining iris mottled metallic green and chocolate brown, more green than brown on dorsal 1/3 and ventral most portions of iris), male with orange nuptial pad at the base of first finger (vs nuptial pads yellowish-gray dorsally, dark gray ventrally); from *A. chanakya* by smaller adult male size, male SVL 56.7–58.6 mm (vs 76.4 mm), supratympanic fold indistinct (vs supratympanic fold strong), the ground coloration of dorsum vivid green, interspersed with irregular black spots (vs dorsal color dull brick-red, spotted with irregular cocoa-brown spots, these cocoa-brown spots enclosing a number of smaller dull brick-red spots), absence of vocal sacs in males (vs vocal sac externally visible); from *A. tawang* by smaller adult male size, male SVL 56.7–58.6 mm (vs 82.5 mm), tibiotarsal articulation of adpressed limb reaching the anterior corner of eye (vs tibio-tarsal articulation reaches to snout), supratympanic fold indistinct (vs supratympanic fold strong), the ground coloration of dorsum vivid green, interspersed with irregular black spots (vs dorsal color olive-green, spotted with large, irregular shaped dark-brown spots, brown spots enclosing a number of small olive-green dots), absence of vocal sacs in males (vs vocal sac faintly visible); from *A. kaulbacki* by smaller body size, male SVL 56.7–58.6 mm and female SVL 63.6–64.8 mm (vs SVL 72.6–82.6 mm in males and 82.7–87.2 mm in females), tibiotarsal articulation of adpressed limb reaching the anterior corner of eye (vs tibio-tarsal articulation reaches to the tip of snout), iris distinctly bicolored, green in upper 1/4 and reddish brown in lower 3/4 (vs iris vivid green with irregular dark patterns), discontinuous glandular dorsolateral fold (vs absent); from *A. viridimaculatus* by smaller body size, male SVL 56.7–58.6 mm and female SVL 63.6–64.8 mm (vs 72.7–82.3 in males and 83.0–94.3 in females), discontinuous glandular dorsolateral fold (vs absent), the ground coloration of dorsum vivid green, interspersed with irregular black spots (vs dorsum and flank with nearly round green or yellowish green spots, scattered with small green spots); from *A. beibengensis* by smaller body size, male SVL 56.7–58.6 mm and female SVL 63.6–64.8 mm (vs SVL 75.8 mm male and 90.2–93.2 mm in females), discontinuous glandular dorsolateral fold (vs absent), male with orange nuptial pad at the base of first finger (vs white nuptial pads), supratympanic fold indistinct (vs distinct, wide and thick); from *A. formosus* by smaller adult female size, SVL 63.6–64.8 mm (vs SVL 79.4–83.7 mm), glandular dorsolateral fold (vs absent), male with orange nuptial pad at the base of first finger (vs developed white nuptial pads); from *A. himalayanus* by smaller body size, male SVL 56.7–58.6 mm and female SVL 63.6–64.8 mm (vs SVL 72.8–76.7 mm males and 80.5–89.0 mm in females), vocal sac absent in males (vs externally visible vocal sacs present), the ground coloration of dorsum vivid green, interspersed with irregular black spots (vs dark brown, interspersed with irregular yellow spots); from *A. pallasitatus* by smaller adult female size, female SVL 63.6–64.8 mm (vs 70.6–72.3 mm in females), glandular dorsolateral fold (vs absent), the ground coloration of dorsum vivid green, interspersed with irregular black spots (vs dorsum yellow-green, with irregular dark brown blotches without margins), rictal gland absent (vs rictal gland prominent and ellipsoidal); from *A. yangi* by larger body size, male SVL 56.7–58.6 mm and female SVL 63.6–64.8 mm (vs SVL 46.3–51.8 mm males and 51.5 mm in female), three metacarpal tubercles, inner metacarpal tubercle long, outer metacarpal tubercle relatively small, oval, median one rounded (vs inner metacarpal tubercle rounded, outer metacarpal tubercle indistinct), a black stripe below edge of the canthus rostralis extending from the nostril across the eyes, along dorsolateral glandular folds to near vent (vs a black stripe below edge of the canthus rostralis extending from the snout tip across the eyes, to the anterior edge of supratympanic fold), toes with narrow lateral fringes, toe IV and postaxial side of toe V relatively wider (vs narrow lateral fringes of preaxial side of toe I and postaxial side of toe V); from *A. longimanus* by larger body size, male SVL 56.7–58.6 mm and female SVL 63.6–64.8 mm (vs SVL 30 mm), eye diameter (ED/SL 70.9%–75.9%) shorter than snout length (vs snout approximately as long as the eye diameter, ED/SL 97.7%), nostrils closer to anterior corner of eye than to tip of snout (vs nostrils a little nearer to tip of snout than the eye), hand length ~ 2/3 of forelimb (vs the hand nearly 1/2 length of the whole forelimb); from *A. medogensis* by smaller body size, male SVL 56.7–58.6 mm and female SVL 63.6–64.8 mm (vs SVL 95.0 mm males and 72.4–96.9 mm in females), supratympanic fold indistinct (vs distinct, wide and thick), tibiotarsal articulation of adpressed limb reaching the anterior corner of eye (vs beyond tip of snout), circummarginal grooves present on discs of outer three fingers, absent on first finger (vs discs of all fingers with circummarginal grooves); from *A. wangyufani* by smaller body size, male SVL 56.7–58.6 mm and female SVL 63.6–64.8 mm (vs SVL 68.3–69.0 mm males and 83.4 mm in female), discontinuous glandular dorsolateral fold (vs absent), tibiotarsal articulation of adpressed limb reaching the anterior corner of eye (vs beyond tip of snout), the ground coloration of dorsum vivid green, interspersed with irregular black spots (vs brown dorsal color, head and body with green markings), male with orange nuptial pad at the base of first finger (vs pale gray nuptial pads); from *A. nidorbellus* by smaller body size, male SVL 56.7–58.6 mm and female SVL 63.6–64.8 mm (vs SVL 76.4–82.3 mm males and 85.4–98.0 mm in females), the ground coloration of dorsum vivid green, interspersed with irregular black spots (vs dorsally brown with small irregularly arranged cobalt green spots), tympanum indistinct (vs distinct).

##### ﻿Etymology

Baihualing, where the new species occurs, lies on the historical passage of the famous Southern Silk Road. The specific epithet *gudao* is derived from the Chinese alternative name for the Southern Silk Road. We propose the English common name Gudao Cascade Frog and the Chinese common name Gǔ Dào Tuān Wā (古道湍蛙).

## ﻿Discussion

The body color and markings within the *A. viridimaculatus* group exhibit significant intraspecific variability. For example, *A. viridimaculatus* shows notable intra-population variation in color pattern. The current *A. viridimaculatus* includes the former *A. splendissimus* and *A. caelumnoctis* which were suggested as junior synonyms of *A. viridimaculatus* based on molecular systematics results (Zhang et al. 2021; Mahony et al. 2022). Consequently, there were significant intraspecific color differences between them. In addition, Hou et al. (2023) demonstrated substantial variation in color patterns among different populations of *A. kaulbacki*. Our study found similar variability in the dorsal markings of *A. gudao* sp. nov. further confirming this observation.

During our field survey in the Gaoligong Mountains, multiple species of *A. viridimaculatus* group were found to be sympatric. *Amolops viridimaculatus* and *A. kaulbacki* were sympatrically distributed in Pianma Town, *A. viridimaculatus* and *A. yangi* in Ega, Lushui County and Yaping, Fugong County, as well as *A. viridimaculatus* and *A. gudao* sp. nov. in Baihualing Village, Mangkuan Town, Longyang. Similar sympatric distribution patterns have also been observed in other amphibians, such as the genus *Leptobrachella* (Ohler et al. 2011; Chen et al. 2020) and *Megophrys* sensu lato (Chen et al. 2017). However, research on the mechanisms underlying the sympatric distribution of amphibians is poorly understood. Future evolutionary studies should integrate life history (e.g., advertisement call and breeding season) with nuclear data (even genome data) to explore the mechanisms of sympatric coexistence among multiple species. In addition, careful attention should be paid to species identification, due to the significant variation in color patterns for interspecies within the *A. viridimaculatus* group and their sympatric distribution.

Since 2019, 31 new species have been described within the genus *Amolops*, accounting for approximately one-third of all recognized species under the genus (Che et al. 2020; Frost 2024). Seventeen of these species, including *A. yangi* Wu, Yu, Lu, Yuan & Che, 2024, *A. ailao* Tang, Sun, Liu, Luo, Yu & Du, 2023, *A. chaochin* Jiang, Ren, Lyu & Li, 2021, *A. dafangensis* Li, Liu, Ke, Cheng & Wang, 2024 and *A. tuanjieensis* Gan, Yu & Wu, 2020, were either discovered or recorded in China (Gan et al. 2020; Tang et al. 2023; Li et al. 2024; Wu et al. 2024a). Our discovery increases the number of *Amolops* species recorded in China to 59 and the total number of *Amolops* species to 86. Furthermore, the discovery of new species in the Gaoligong Mountains further proves that the amphibian diversity in this mountain ecosystem has been underestimated, underscoring the need for continued fieldwork and enhanced research efforts.

## Supplementary Material

XML Treatment for Amolops gudao

## ﻿Additional information

### Author ORCIDs

Jing Che https://orcid.org/0000-0003-4246-6514

### Data availability

All of the data that support the findings of this study are available in the main text or Supplementary Information.

## Supplementary materials

Supplementary material 1 Localities, voucher information, and Genbank accession numbers for all specimens used in this study Data type xls Explanation note The “*” indicates that the sequences are derived from the holotype or paratype. https://binary.pensoft.net/file/1247844This dataset is made available under the Open Database License (http://opendatacommons.org/licenses/odbl/1.0/). The Open Database License (ODbL) is a license agreement intended to allow users to freely share, modify, and use this Dataset while maintaining this same freedom for others, provided that the original source and author(s) are credited.Yun-He Wu, Zhong-Bin Yu, Felista Kasyoka Kilunda, Chen-Qi Lu, Jia-Hua Li, Yun-Peng Li, Yi-Juan Shi, Jing Che

Supplementary material 2 Average uncorrected p-distances among the *Amolops viridimaculatus* group individuals calculated from 16S rRNA gene sequences Data type xls https://binary.pensoft.net/file/1247845This dataset is made available under the Open Database License (http://opendatacommons.org/licenses/odbl/1.0/). The Open Database License (ODbL) is a license agreement intended to allow users to freely share, modify, and use this Dataset while maintaining this same freedom for others, provided that the original source and author(s) are credited.Yun-He Wu, Zhong-Bin Yu, Felista Kasyoka Kilunda, Chen-Qi Lu, Jia-Hua Li, Yun-Peng Li, Yi-Juan Shi, Jing Che

Supplementary material 3 Selected diagnostic characters for species described herein and species in *Amolops viridimaculatus* group Data type xlsx Explanation note The morphological characters are based on mainly the following literature: Andersson 1938; Biju et al. 2010. https://binary.pensoft.net/file/1560551This dataset is made available under the Open Database License (http://opendatacommons.org/licenses/odbl/1.0/). The Open Database License (ODbL) is a license agreement intended to allow users to freely share, modify, and use this Dataset while maintaining this same freedom for others, provided that the original source and author(s) are credited.Yun-He Wu, Zhong-Bin Yu, Felista Kasyoka Kilunda, Chen-Qi Lu, Jia-Hua Li, Yun-Peng Li, Yi-Juan Shi, Jing Che
